# Proteomic Analysis of MG132-Treated Germinating Pollen Reveals Expression Signatures Associated with Proteasome Inhibition

**DOI:** 10.1371/journal.pone.0108811

**Published:** 2014-09-29

**Authors:** Candida Vannini, Marcella Bracale, Rita Crinelli, Valerio Marconi, Paola Campomenosi, Milena Marsoni, Valeria Scoccianti

**Affiliations:** 1 Dipartimento di Biotecnologie e Scienze della Vita, Università degli Studi dell'Insubria, Varese, Italy; 2 Dipartimento di Scienze Biomolecolari, Università di Urbino “Carlo Bo”, Urbino, Italy; 3 Dipartimento di Scienze della Terra, della Vita e dell'Ambiente, Sezione di Biologia Vegetale, Università di Urbino “Carlo Bo”, Urbino, Italy; St. Georges University of London, United Kingdom

## Abstract

Chemical inhibition of the proteasome has been previously found to effectively impair pollen germination and tube growth *in vitro*. However, the mediators of these effects at the molecular level are unknown. By performing 2DE proteomic analysis, 24 differentially expressed protein spots, representing 14 unique candidate proteins, were identified in the pollen of kiwifruit (*Actinidia deliciosa*) germinated in the presence of the MG132 proteasome inhibitor. qPCR analysis revealed that 11 of these proteins are not up-regulated at the mRNA level, but are most likely stabilized by proteasome inhibition. These differentially expressed proteins are predicted to function in various pathways including energy and lipid metabolism, cell wall synthesis, protein synthesis/degradation and stress responses. In line with this evidence, the MG132-induced changes in the proteome were accompanied by an increase in ATP and ROS content and by an alteration in fatty acid composition.

## Introduction

Proteolysis is a key mechanism regulating protein homeostasis in living cells. The ubiquitin/proteasome pathway (UPP) is the most important proteolytic system in eukaryotic cells and is essential for protein quality control because it selectively eliminates misfolded, damaged and oxidized proteins. Since the accumulation of altered polypeptides may become cytotoxic, UPP failure may have severe consequences for cellular homeostasis and survival. Even more crucial, the UPP is an important regulatory tool of the cell that controls the concentrations of numerous signaling mediators and transcription factors [1], [2]. In plants, selective UPP-mediated protein degradation is critical for many key processes such as hormone perception and signaling, light response, flower development, self-incompatibility, epigenetic regulation, stress response and disease control [2]–[7].

Pollen germination and tube growth are essential for higher plant sexual reproduction. These processes are highly dynamic and coordinated, integrating many internal and external environmental signals in both space and time [8], [9]. Transcriptomic and proteomic data suggest that quick pollen germination and early tube growth largely depend on presynthesized mRNA and protein [10]. On the other hand, protein turnover seems to be essential during tube organization and elongation and several observations have provided evidence that in this context the UPP system is the major regulator. In particular, the upregulation of six subunit components of the 20S core complex of the 26S proteasome has been reported to occur during pollen tube growth [10]. Furthermore, Arabidopsis mutants in regulatory particles of proteasome display alterations in pollen development [11], [12]. Finally, chemical proteasome inhibitors impair pollen tube emergence and growth severely and induce aberrant morphology in *Picea wilsonii* and *Actinidia deliciosa* [13]–[16]. In particular, treatment with benzyloxycarbonyl-leucinyl-leucinyl-leucinal (MG132) caused cytoskeleton disorganization, accompanied by cytoplasmic streaming inhibition and loss of polarized distribution of organelles and nuclei [13], [16]. These findings suggest that the ubiquitin proteolytic pathway may control the levels of regulatory proteins essential for cytoskeleton organization. In addition, following proteasome inhibition, a marked decline in cell wall components (pectins and cellulose) was observed especially in tube apical and subapical regions [13]. It has been hypothesized that inhibitor-induced cytoplasmic vacuolization and weakening of cell walls could be responsible for tip swelling and irregularly broadened tube diameters. More recently, it has also been reported that MG132 strongly disturbs mitochondrial remodeling and reduces mitochondrial membrane potential during pollen tube development in *Picea wilsonii* [14]. Finally, Qiao et al. [17] revealed that MG132 specifically blocks compatible pollination in *Antirrhinum deliciosa* by inhibiting degradation of non-self S-RNases.

While these studies have focused mainly on the morphological and cytological effects of UPP malfunction during pollen germination, little is known about molecular mechanisms affected by proteasome inhibition. In the present study, the effect of proteasome inhibitor MG132 on *Actinidia deliciosa* pollen germination was investigated by combining two-dimensional electrophoresis (2-DE) and liquid chromatography-electro spray tandem mass spectrometry (LC-ESI-MS/MS) analysis with the aim of identifying proteins whose abundance and/or activity are directly/indirectly affected by the ubiquitin-proteasome proteolytic pathway. ATP content, lipid profile and ROS production were also analyzed, based on the predicted function of the differentially expressed proteins. The results of these studies are expected to contribute to the shedding of light on the nature and regulation of molecular players crucial for pollen germination and tube growth. The factors that control these processes are, in fact, of considerable interest to the understanding of plant fertility and reproduction mechanisms, as well as in the development of molecular tools aimed at manipulating pollen tube growth for practical purposes.

## Materials and Methods

### Plant material

Pollen of male kiwifruit *Actinidia deliciosa* var.deliciosa (A. Chev.), C.F. Liang et A.R. Ferguson] was obtained from plants of cv Tomuri growing in experimental plots at the Azienda Tarozzi near Faenza (Italy). Anthers, collected from central flowers of the three-six-flowered inflorescence, were allowed to dehisce under controlled conditions as described by Calzoni et al. [18]. Pollen was separated by sieving and stored at −20°C until use.

The percentage of viable pollen grains before freezing was 94±1%, as determined using the fluorochromatic reaction with fluoresceine diacetate which assesses the integrity of the plasmalemma of the vegetative cell [19]. Compared to fresh pollen of the same year no significant decline in viability was registered during the lasting of the experiments described in this paper.

### In vitro pollen germination

Pollen was rehydrated for 30 min at 30°C under 100% relative humidity. Germination was performed by suspending the rehydrated pollen grains (1 mg mL^−1^) in a liquid medium containing 0.29 M sucrose and 0.4 mM boric acid according to Scoccianti et al. [16]. Proteasome inhibitor MG132 (Biomol Research Laboratory, Plymouth Meeting, PA) was added into the medium at the beginning of incubation to a final concentration of 40 µM (MG132-treated). Since the inhibitor was dissolved in dimethylsulphoxide (DMSO), parallel incubations containing the solvent alone, at the same concentration (0.08%) that was present in MG132-treated samples, were set up (DMSO-controls). In some cases, pollen cultures receiving neither MG132 nor DMSO (controls) were run in parallel. Cultures were incubated for 90 min, unless otherwise specified, at 30°C in the dark. After incubation, pollen cultures were centrifuged at 1400 *g* for 2 min, the supernatants were discarded and the cells were washed with fresh medium containing 0.29 M sucrose. Cells were then collected by Millipore vacuum filtration (Millipore Corporation, Billerica, MA), detached from the filter membrane (8.0 µm pore size) and immediately used for the subsequent analyses or frozen in liquid nitrogen and stored at −80°C until use.

The percentage of tube emergence (% germination) was determined with an image analyzer-Axioplan microscope (Zeiss, Jena, Germany) combination by scoring at least 1000 randomly chosen pollen grains per sample, resulting from the sum of several non overlapping fields. Pollen was considered germinated only when the tube length was greater than the diameter of the grain.

### Protein sample preparation

Samples were homogenized by using mortar and pestle in liquid nitrogen with an addition of sand quartz. Total proteins were extracted as described by Marsoni et al. [20]. After sample clarification at 13000 *g* for 10 min, the protein concentration was measured by Bio-Rad protein assay (Hercules, CA, USA), using bovine serum albumin as a standard. The samples were directly loaded for isoelectrofocusing (IEF) or stored in aliquots at −80°C until use. The extractions were performed in triplicate and the results were highly reproducible.

### Two-dimensional IEF/SDS-PAGE

IEF was carried out with 700 µg of total protein extract by using an immobilized 4 to 7 pH gradient (Immobiline DryStrip, 18 cm; Amersham Biosciences, Uppsala, Sweden). The strips were rehydrated in the IPGphor system (Amersham Biosciences, Bucks, UK) for 1 h at 0 V, 20°C and 10 h at 30 V, 16°C with the solubilization buffer containing 7 M urea, 2 M thiourea, 4% 3-[(3-Cholamidopropyl) dimethylammonio]-1-propanesulfonate (CHAPS), 50 mg/mL dithiothreitol (DTT), 0.5% of carrier ampholyte (3–10 NL IPG buffer; Amersham Biosciences), bromophenol blue 0.005% and the protein extracts. IEF was performed at 16°C in the IPGphor system (Amersham Biosciences) for 4 h at 200 V, from 200 to 3500 V in gradient during 30 min, 3 h at 3500 V, from 3500 to 8000 V in gradient during 30 min, after which the run was continued at 8000 V to give a total of 70 kVh.

Each focused strip was equilibrated for 30 min against 6 M urea, 30% glycerol, 2% SDS, 50 mM Tris-HCl pH 8.8, 2% DTT and then a further 30 min with the substitution of the DTT with 2.5% iodoacetamide in the equilibration buffer. The equilibrated strips were placed on top of vertical 12.5% polyacrylamide gels (acrylamide/PDA 12.5% T, 2.6% C, 0.375 M Tris-HCl pH 8.8, TEMED 0.05% v/v, APS 0.1% w/v). The molecular weight markers, covering a 10 to 250 kDa range, were run on the acidic side of each gel. Electrophoresis was performed at 4°C in a Laemmli running buffer (25 mM Tris-HCl pH 8.3, 192 mM glycine, 0.1% SDS) for 30 min at 15 mA/gel then at 45 mA/gel until the dye front reached the bottom of the gel. Each extraction was analyzed by three gel replicates.

### Staining and analysis of 2DE gels

Proteins were detected with Colloidal Coomassie Brilliant Blue (CCBB) modified as described in Marsoni et al. [21]. Thereafter, gels were digitalized by ImageScanner (Amersham Bioscience). Image and data analysis of the scanned (300 dpi, 16-bit greyscale pixel depth) gels as TIFF files were performed by using the Image Master 2D Platinum imaging software version 5.0, which allows spot detection, spot matching among multiple gels, background subtraction and spot area and optical intensity quantization. For this purpose, data were normalized by expressing protein abundance as percent spot volume relative to volume of total protein in the gel (%Vol). Three replicate gels in each experimental group were averaged and the resulting gels (average-gels) contained only spot presents in all the replicates. The average-gels were compared and only proteins with a fold change of ±1.5, significant in Student's *t*-test at a level of 95%, were accepted as differentially expressed. These spots were selected for MS/MS analysis.

### Mass Spectrometry analysis and protein identification

Spots digestion was performed as described in Marsoni et al. [21]. The extracted tryptic fragments were analysed by MS/MS after reverse phase separation of peptides (liquid chromatography-electro spray tandem mass spectrometry, LC-ESI-MS/MS). For all experiments, a Finnigan LXQ mass spectrometer, equipped with a Finnigan Surveyor MS Pump Plus HPLC system (Thermo Electron Corporation, CA, and USA), was used. Chromatography separations were conducted on a BioBasic C18 column (150 µm I.D.×150 mm length and 5 µm particle size; Thermo Electron Corporation, USA), using a linear gradient from 5 to 75% acetonitril (ACN), containing 0.1% formic acid with a flow of 2 µL min^−1^. Including the regeneration step, one run lasted 80 min. Acquisitions were performed in the data-dependent MS/MS scanning mode (full MS scan range of 400–1400 m z^−1^ followed by Zoom scan for the most intense ion from the MS scan and full MS/MS for the most intense ion from the zoom scan), thus enabling a dynamic exclusion window of 3 min.

Protein identifications were conducted by correlation of uninterpreted tandem mass spectra to the entries of a non-redundant protein and/or EST-viridiplantae database downloaded from the National Center for Biotechnology Information (NCBI NR 04/29/2011: 13841106 sequences, 4750259772 residues. Viridiplantae: 881708 sequences. Plants_EST: 144355992 sequences, 25218209504 residues) using the MASCOT open source (http://www.matrixscience.com). The parameters were set to allow one missed cleavage and considering as variable modification cysteine carbamido-methylation and methionine oxidation. The precursor ion tolerance was set at 1.2 Da and fragment ion tolerance 0.6 Da. Only proteins with a minimum of two matching peptides were considered and Mowse scoring system was used to assign correct identification.

### RNA extraction and qPCR analysis

Total RNA from pollen was extracted with the Spectrum Plant Total RNA Kit (Sigma, Milan, Italy), according to the manufacturer's instructions and treated with DNase 1 (AMPD, Sigma, Milan, Italy). For real-time quantitative PCR (qPCR), cDNA was generated from 1.5 µg of RNA by using the iScript cDNA synthesis kit (Biorad, Milan, Italy). qPCR was performed on an AB7000 thermal cycler (AB, Milan, Italy) using the iTAQ Universal Sybr Green Supermix (Biorad, Milan, Italy). The total RNA was extracted twice (in two independent experiments). From each extraction two independent cDNA were prepared to minimize the impact of random fluctuations. Three amplifications were subsequently performed for each relevant gene and all amplifications were repeated on a second independent plate. Relative mRNA quantification was obtained using the ΔΔCt method, using Actin and 18S rRNA as reference. Tubulin was not used as reference as its expression was found affected by the treatment. Melting curve analysis was performed to ensure that single amplicons were obtained for each target. Primers for the genes under investigation were designed to encompass at least one intron, whenever possible. To do this, for each protein identified by Mascot, the full length or partial cDNA clone from Actinidia (for which no information on genomic DNA is available) was retrieved and subjected to Blast analysis to obtain the sequence of the nearest ortholog in a species for which genomic information was available. The mRNA-to-genomic alignment program “Spidey”, available at NCBI (http://www.ncbi.nlm.nih.gov/spidey/), was used to obtain information on gene organization, assuming that intron-exon boundaries would be conserved among the species under consideration. This information was used to set the parameters for designing the primers using the Primer-Blast tool at NCBI (http://www.ncbi.nlm.nih.gov/tools/primer-blast/). Data on Blast analyses are provided on Table S1; primer sequences are reported in Table S2.

### Phosphoglycerate kinase enzymatic assay

Samples were lysed in liquid nitrogen by four freeze-thaw cycles in the presence of 50 mM Tris-HCl, pH 7.8 containing a cocktail of proteasome inhibitors (Roche Applied Science, Indianapolis, IN, USA). The extract was tip sonicated on ice for 15 sec at 50 W, three times. Sonication was performed with a Labsonic 1510 Sonicator (Braun, Melsun- gen, Germany) equipped with a 4 mm probe. Samples were then centrifuged at 12000 rpm in an Eppendorf centrifuge for 10 min at +4°C to remove insoluble debris. Enzyme activity was assayed spectrophotometrically by monitoring the oxidation of NADH at 340 nm, according to Beutler [22].

### ATP content determination

Samples were immediately extracted in cold 7% perchloric acid using a Potter–Elvehjem apparatus. The extracts were centrifuged at 14000 *g* for 10 min at 4°C. Aliquots of the supernatants (500 µL) were neutralized with 3 M K_2_CO_3_ solution and centrifuged at 5000 *g* for 10 min at 4°C. The ATP content was determined spectrophotometrically following the reduction of NADP^+^ to NADPH at 340 nm, as reported by Beutler [22].

### Dosage of lipids and fatty acid profile analysis

Total lipid content was determined in kiwifruit pollen after 0, 30, 60 or 90 min of incubation, in the absence (DMSO-control) or in the presence of 40 µM MG132, according to the method of Izard and Limberger [23]. Pollen cultures were centrifuged at 1400 *g* for 2 min, the supernatants were discarded and the cells were suspended with MilliQ water and lysed in liquid nitrogenby five freeze-thaw cycles. One-hundred microliter aliquots of the lysed cell suspensions, or of MilliQ water (reference tube), were added to a stoppered glass tube. Two milliliters of 96% sulphuric acid were then added to each tube. The tubes were incubated in a boiling water bath for 30 min and then cooled for 5 min in a water bath at room temperature. Five milliliters of phosphoric acid–vanillin reagent [0.6 g of vanillin (4-hydroxy-3-methoxybenzaldehyde; Sigma) in 100 mL distilled water, with the volume adjusted to 500 mL with 85% phosphoric acid] were added to the tubes and incubated at room temperature for 15 min, and then absorbance was read at 530 nm. The reference curve was composed of Triolein (1,2,3-tri[*cis*-9-octadecenoyl] glycerol; Sigma) ranging from 10 to 100 µg [23].

Fatty acids (FAs), extraction was carried out according to the method of Folch et al. [24] with few modifications. Samples were extracted with a chloroform/methanol solution (2∶1, v∶v) under stirring for 1 min, and then equilibrated with one fourth its volume of distilled water. Extracts were centrifuged for 20 min at 3500 *g*. The upper phase was discarded and the lower was filtered through a 0.2 µm pore PVDF membrane. The filtrate was collected and taken to dryness in a rotary evaporator. Methyl esters of fatty acids were obtained suspending the samples in 100 µL of 2 M KOH-CH_3_OH with shaking for 10 min and then extracted with 100 µL of n-hexane by stirring with a vortex for 30 sec at maximum speed. The hexane phase, containing fatty acid methyl esters, was transferred into a clean tube, and the residual phase was extracted again with 100 µL of n-hexane. The hexane phases were combined and concentrated with a stream of liquid nitrogento a final volume of 50 µL. For gas chromatography/mass spectrometry analysis of fatty acid methyl esters, a gas chromatograph (Agilent 6890 N, U.S.A.), coupled with double focalization high resolution Jeol JMS-GC-Mate II (Japan) in full scan mode and equipped with Jeol Software, was used. Chromatographic separation was performed using a Supelcowax 10 GC column (0,25 mm i.d.×30 m length, 0,25 µm film thickness). The oven temperature was programmed as follows: 100°C, hold for 3 min, 20°C min^−1^ to 165°C no hold, 12°C min^−1^ to 270°C, hold for 5 min. The carrier gas was helium with a constant flow of 1 mL min^−1^. The injector temperature was 260°C and the interface temperature was 270°C. Injection volume was 1 µL with 3 min of delay time in splitless mode.

### ROS production

The nitroblue tetrazolium (NBT) assay described by Wang et al. [25] was used, with slight modifications concerning incubation time and temperature. After 30 and 90 min of incubation in the absence (control) or in the presence of 0.08% DMSO (DMSO-control) or 40 µM MG132, pollen cultures were centrifuged at 1400 *g* for 2 min. and the pelletted cells were added with 2 mM NBT. After 30 min of the reaction at 30°C, the suspensions were centrifuged and washed twice with 1 mL water. The formazan produced by NBT reduction was dissolved in 1 mL of methanol and incubated for 30 min at 30°C. After centrifugation, the absorbance of supernatants was read at 530 nm.

### Detection of carbonylated protein

Protein carbonylation assay was assessed by using the commercially available OxyBlot protein oxidation detection kit (Millipore). Protein samples containing 40 µg of proteins were added to an equal volume of 12% SDS. Then protein carbonyl groups were derivatized to 2, 4-dinitrophenylhydrazone (DNP) by incubation with one additional volume of 2,4-dinitrophenylhydrazine for 30 min at room temperature.

The derivatization reaction was stopped by the addition of 20 µL neutralization solution. Proteins were separated by 12% SDS-PAGE and transferred to PVDF membrane (Serva). The oxidatively modified proteins were detected using anti-DNP antibodies (anti-dinitrophenyl-group antibodies, Sigma) and visualized by a chemiluminescence detection kit (SuperSignal, Pierce Biotechnology, Rockford, IL, USA). To monitor the equal loading of samples, CCBB was used to stain the proteins in a duplicate gel.

### Statistical analysis

Statistical analysis of data was performed by ANOVA for repeated measurement, followed by the Tukey-Kramer multiple comparison test (for n groups>2) or with the paired t-test (for n groups = 2), using the GraphPad Prism package, version 5.0 for Macintosh (GraphPad Software, San Diego, CA, USA). Differences between values were assumed statistically significant at p<0.05 (*), very significant at p<0.01 (**).

## Results and Discussion

### Proteasome inhibition alters the expression of soluble proteome

To investigate the protein changes induced by proteasome inhibition, kiwifruit pollen was cultured in the presence of 40 µM MG132 for 90 min. Doses of MG132 ranging from 10 to 100 µM have been used to analyze the involvement of the proteasome in various aspects of plant physiology [26]–[28]. These differences probably reflect a different sensitivity of the cells/organisms and/or of the proteasome inhibition pathway studied; depending on the experimental model investigated, different uptake and toxicity of inhibitors could be also suggested. Concerning pollen germination, concentrations of MG132 between 20 and 80 µM have been shown to be effective in altering pollen germination and tube length in *Picea wilsonii* with a dose-dependent effect particularly evident between 10 and 40 µM, at least as shown for tube elongation [13]. Similarly, we have previously demonstrated that kiwifruit pollen germination is affected by treatment with both 40 and 80 µM MG132. In particular, the tube mass produced and the rate of tube elongation were altered in a dose-dependent manner [15], [16]. Notably, at 40 µM MG132, kiwifruit pollen germination and tube growth were markedly affected, but were not almost completely blocked, as at the higher dose of 80 µM. Thus, in light of these findings and with the aim of limiting indirect versus MG132-induced direct effects, all the analyses performed in this study were conducted using MG132 at the final concentration of 40 µM.

**Figure 1:**
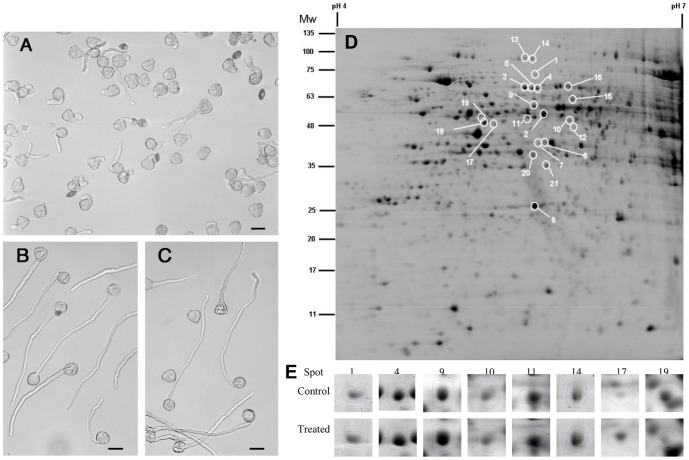
Effects of MG132 on kiwifruit pollen. Pollen after 90 min of incubation in the presence of 40 µM MG132 (A), 0.08% DMSO (B), or without any treatment (C). Bar = 30 µm. D) Representative gel of MG132-treated pollen showing significant (*p*<0.05) changes in protein abundance. (E) Selected differentially accumulated protein spots in pollen exposed to 40 µM MG132.

Under these experimental conditions, the percentage of germination was 87.3±3.8%, 84.6±2.5% and 54.0±4.9% in control, DMSO-control and MG132-treated pollen, respectively. Therefore, MG132 induces a reduction in pollen germination of more than 30% compared to DMSO-control or untreated pollen. It is worth noting that we previously demonstrated that the effect of MG132 was reversible, indicating that the inhibition of pollen germination was not due to cell death [15]. Moreover, in the presence of MG132, the majority of germinated pollen grains produced markedly shorter tubes with altered morphology (Figure 1A). In particular, tubes showed diffuse tip swelling and branching. No solvent effect due to 0.08% DMSO was evident comparing both tube length as well as morphology of DMSO-controls (Figure 1B) and controls (Figure 1C) in agreement with our previous results [15].

To detect changes in the proteome, total soluble proteins from MG132-treated and DMSO-control pollen were analyzed by using both 2DE and a mass spectroscopy approach. We extracted 35.5±3.5 µg and 34.3±5.5 µg of proteins from 1 mg of DMSO-control and MG132-treated pollen respectively. Following CCBB staining, approximately 600 reproducible protein spots were detected per sample (Figure 1D). All spots were matched by gel-to-gel comparisons and the differences in the relative abundance (% vol) of each spot were evaluated by software-assisted analysis. The ANOVA test (*p*<0.05), coupled with a threshold of 1.5-fold change in levels, clearly revealed 24 differentially expressed protein spots in samples treated with MG132 compared with respective control, as shown in Figure 1D, E. These differentially expressed proteins mainly accumulated under conditions of proteasome inhibition, while only one protein (spot 19) was downregulated in MG132-treated vs. DMSO-control pollen.

The excised spots were in-gel digested and analyzed by LC-ESI-MS/MS; 22 protein spots were successfully identified, corresponding to 14 unique proteins (Table S3). The experimental molecular masses (MW) and pIs of the majority of the identified proteins were consistent with the theoretical values, as judged by the locations of the spots on 2-D gels. However, there were some exceptions: spots 10, 11, 12, 6 and 7 displayed more acidic experimental pI values than the theoretical values, while spot 1 had a higher experimental MW than the MW of the corresponding identified protein (Table S3). The uniquely identified proteins were categorized as follows into five functional groups based on their predicted function: energetic metabolism (4), lipid metabolism (3), cell wall synthesis (1), protein synthesis/degradation (4) and stress response (2).

**Figure 2:**
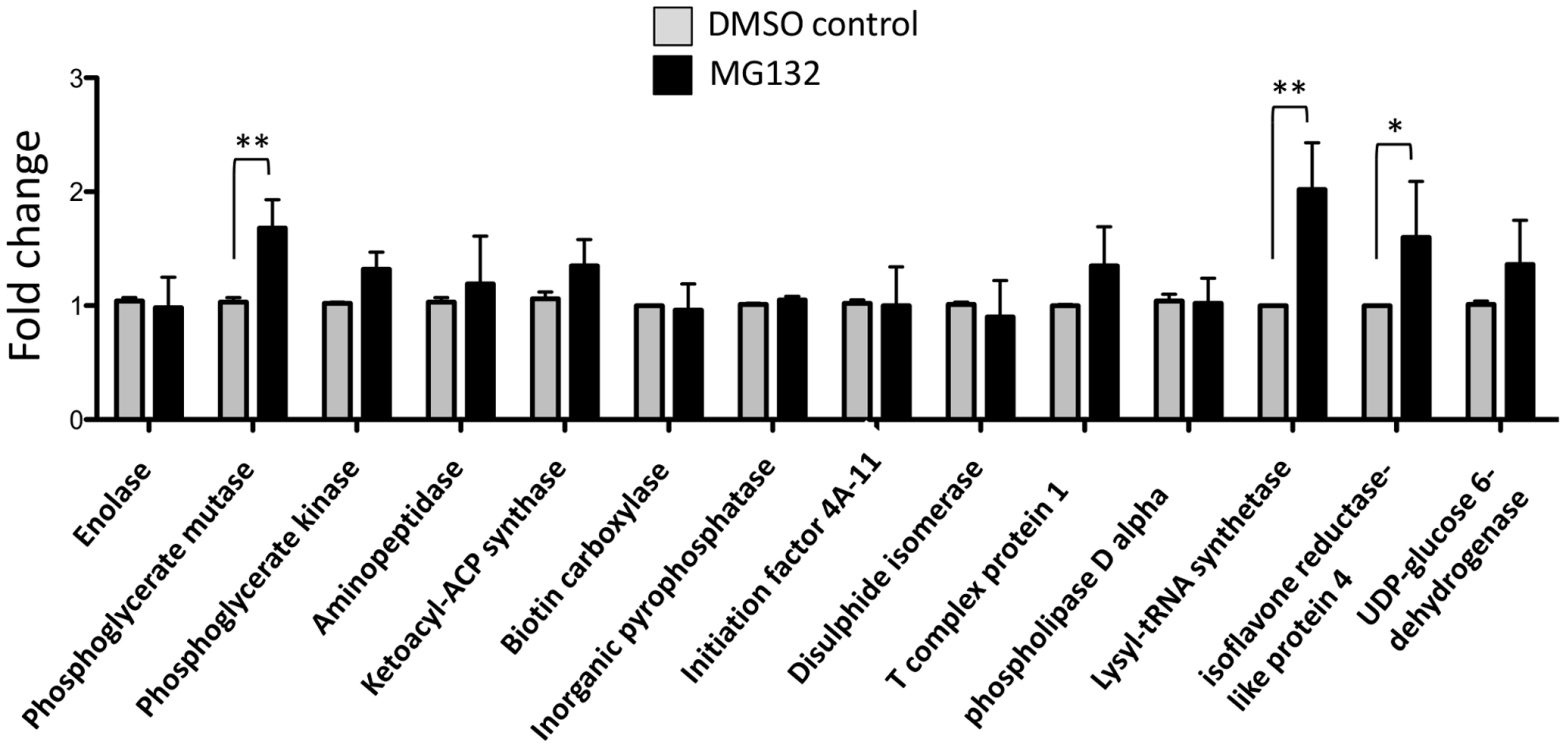
qPCR analysis of DMSO-control or MG132-treated kiwifruit pollen after 90 min of incubation. The values reported are means ± SD of two independent experiments and several technical replicates. Asterisks indicate statistically significant differences in MG132-treated samples relative to DMSO-controls (* p<0.05; ** p<0.01).

To investigate altered protein level mechanisms, we analyzed the RNA expression of genes corresponding to the differentially accumulated proteins by qPCR. The results showed that the observed accumulation of these proteins in pollen treated with MG132 was primarily due to altered protein turnover rather than altered regulation of mRNA levels (Figure 2). Only the levels of phosphoglycerate mutase, lysyl-t-RNA synthase and isoflavone reductase transcripts showed a statistically significant increase in pollen treated with MG132 compared to the DMSO-control (Figure 2).

### Energy metabolism

Treatment with MG132 induced the accumulation of four unique proteins involved in energy metabolism. Three of these, namely phosphoglycerate kinase (PK), phosphoglycerate mutase (PGM) and enolase (ENO), act consecutively in the glycolytic pathway to produce phosphoenolpyruvate (PEP), which is indispensable for energy metabolism. The qPCR analysis indicated that the accumulation of PGM was associated to increased transcript levels (Figure 2), suggesting that proteasome inhibition could indirectly affect the intracellular levels of the enzyme.

**Figure 3:**
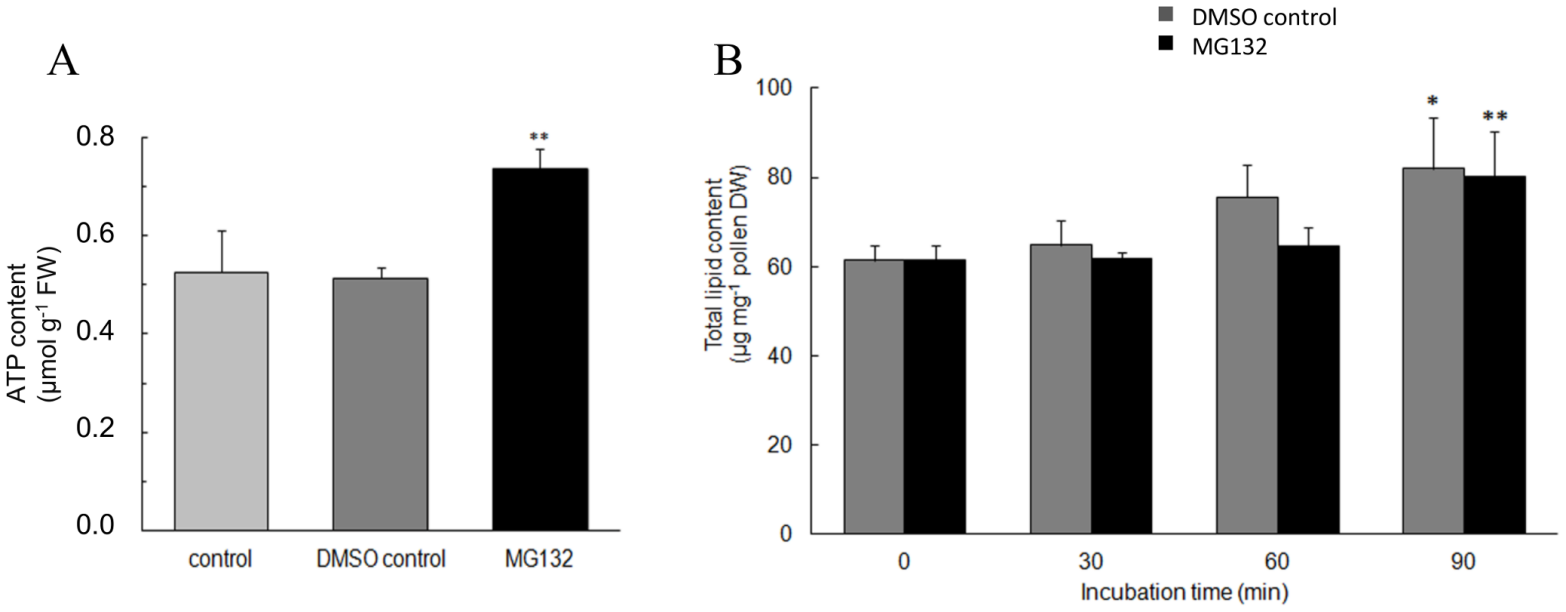
ATP and total lipid content. A) ATP content in control, 0.08% DMSO-control or 40 µM MG132-treated kiwifruit pollen after 90 min of incubation. Asterisks indicate statistically significant differences relative to controls. FW, fresh weight. B) Lipid content at different times of germination in the presence of MG132 or 0.08% DMSO. Asterisks indicate statistically significant differences compared with the respective time zero. DW, dry weight. All data are the means ± SD of two independent experiments each with three biological replicates.

Since the proteomic data indicate that there was an accumulation of enzymes involved in energy metabolism, we evaluated the effect of MG132 on ATP content in kiwifruit pollen after 90 min of incubation. As shown in Figure 3A, a 43% increase in ATP content was observed in MG132-treated pollen compared to DMSO-control pollen. However, when PK activity was assayed no significant differences were detected between MG132- and DMSO-control pollen (1.38±0.16 vs 1.42±0.14 U/mg of total proteins, respectively). This evidence together with the fact that the pI of spots corresponding to PK showed a strong shift toward more acidic values, suggests that the protein accumulates in an oxidized inactive form. Finally, as far as ENO is concerned, the observed MW of the spots was higher than the theoretical value, suggesting possible ubiquitination of this protein. In light of this evidence, it could be hypothesized that the accumulation of ATP in MG132-treated pollen might depend on lower usage rather than on increased production. This is because, pollen tube growth is critically dependent on ATP pools which are used to power energy-consuming processes such as actin polymerization and exo- and endocytosis [29] and following MG132 treatment these processes are inhibited [13].

### Lipid metabolism

In MG132-treated pollen, we detected the accumulation of three unique proteins involved in fatty acid metabolism: an acetyl-CoA biotin carboxylases (BC), a ketoacyl-ACP synthase (KAS I) and a phospholipaseDα (PLDα) (Table S3). BC is a subunit of heteromeric acetyl-coenzyme carboxylase (htACCase), which produces malonyl-CoA in plastids and is a major regulatory point for plant fatty acid synthesis. Madoka et al. [30] showed that post-transcriptional regulation is involved in the formation of a functional htACCase. Moreover, KAS I catalyzes the elongation of *de novo* synthesised fatty acids and is therefore crucial for fatty acid synthesis in Arabidopsis [31]. In the presentexperimental system, the accumulation of BC and KAS I proteins in MG132-treated pollen may affect the biosynthesis of fatty acids required for normal pollen tube development.

To test whether proteasome inhibition resulted in an altered lipid metabolism, we investigated total lipid content and fatty acid composition. In both MG132-treated and DMSO-control pollen, we observed a statistically significant increase in lipid content after 90 min of germination. However, no differences were found between DMSO-control and proteasome inhibitor-treated cells at all time points considered (Figure 3B).

**Figure 4:**
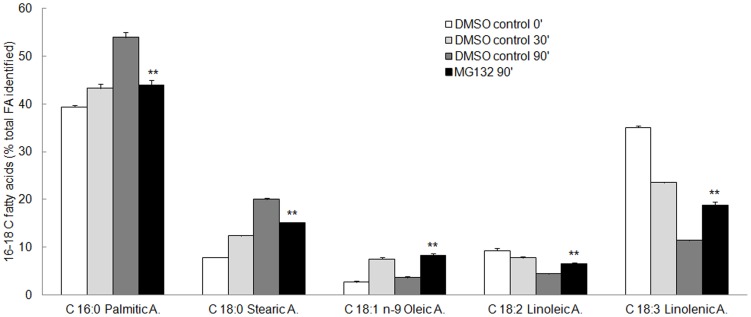
Fatty acid profile in kiwifruit pollen at different times of incubation in the presence of 0.08% DMSO (DMSO-control) or 40 µM MG132. Each fatty acid was identified by comparing its fragmentation pattern with the mass spectrometer database. Results of triplicate analysis were averaged and the content of individual fatty acids in pollen was expressed as percentages of the total fatty acids identified ± S.D. Asterisks indicate significant differences compared with the respective DMSO-control. In some cases bars are too close to be visible. FA, fatty acids.

The following fatty acids were separated and identified in kiwifruit pollen: palmitic acid (C16:0), stearic acid (C18:0), oleic acid (C18:1, n-9), linoleic acid (C18:2, n-6), linolenic acid (C18:3) (Figure 4). Similar results were obtained in pollen from other species [32], [33]. Small quantities of eicosanoic acid (C20:0) (2.98%), eicosadienoic acid (C20:2) (0.6%), eicosatrienoic acid (C20:3) (0.68%) and docosanoic acid (C22:0) (2.05) were also detected.

After 90 min of incubation in the presence of MG-132 the content of palmitic acid was lower than that found in the respective DMSO-control, suggesting that KAS I, used for making 6–16 C fatty acids, accumulates in an inactive form. Significantly, the isoelectric point of the accumulated protein was more acidic than that of the native form, possibly as a consequence of oxidative damage (Table S3). From the fatty acids profile it was also evident that upon MG132 treatment, a higher amount of the unsaturated fatty acids oleic, linoleic and linolenic acids was detected (Figure 4). From these data it is possible to speculate that proteasome modulates the activity of desaturating enzymes. It is worth noting that during germination, a time-dependent increase in saturated palmitic and stearic fatty acids occurs together with a concomitant decrease in unsaturated linoleic and linolenic acids (Figure 4). Thus, the alteration in fatty acid composition associated with proteasome inbition may contribute in explaining the impairement of pollen germination and tube growth.

The secretion of cell wall polysaccharides in growing pollen tips also requires spatial and temporal coordination of secretory vesicle trafficking. In MG132-treated pollen, we detected the accumulation of a PLDα (Table S3). PLD hydrolyzes common structural phospholipids to produce phosphatidic acid (PA). PLD and PA produced by PLD are involved in the production of exocytotic vesicles, in their delivery to the pollen tube apex and in their fusion with the plasma membrane [34], [35]. In pollen tubes, the majority of PA is produced via the PLD pathway, and the expansion of the apical region of the pollen tube is associated with a several-fold increase in PA content [36]. Moreover, Pleskot et al. [35] demonstrated that actin polymerization is specifically regulated by PLD-derived PA in tobacco pollen tubes. These data suggest the proteasome exerts a regulatory effect on pollen tube development through the modulation of phospholipid signaling.

### Cell wall biosynthesis

Pollen tubes are tip-growing cells and their growth requires the continuous deposition of cell wall material in a highly controlled manner to ensure the morphogenesis of a perfectly cylindrical structure. MG132-treated pollen exhibits dramatic modifications in tube-tip morphology, tip swelling and irregularly broadened diameters [16], [13].

In pollen treated with MG132, we identified the accumulation of UDP-glucose dehydrogenase (UGD) (Table S3), an enzyme that oxidizes the conversion of UDP-D-glucose to UDP-D-glucuronate, a key sugar nucleotide involved in the biosynthesis of plant cell wall polysaccharides [37]. UGD is a good candidate for a control point in the metabolic pathway of cell wall synthesis because it is present at low concentrations with respect to other enzymes in the pathway and it operates far from equilibrium [38]. UGD homeostasis is highly important for cell wall integrity. In fact, both over-expression and suppression of this enzyme reduce the polysaccharide concentrations of the cell wall [39]–[42]. The altered homeostasis of UGD in the presence of MG132 correlates very well with the observed inhibition of pollen tube growth and with the morphological disorder of the pollen tube. Our data confirm the hypothesis of Sheng et al. [13] that MG132 inhibits the turnover of enzymes associated with cell wall organization.

### Stress response

**Figure 5:**
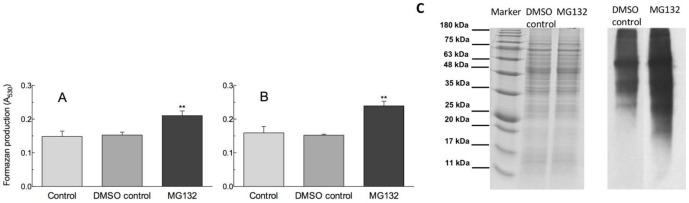
ROS levels and oxidatively modified proteins. ROS generated by germinating kiwifruit pollen after 30 (A) or 90 min of incubation (B) in the absence (control) or in the presence of DMSO (DMSO-control) or MG132. Data are mean values ± S.D. of three replicates. Asterisks indicate significant differences relative to controls. C) Protein stain (left) and anti-DNP immunoassay (right) are shown.

MG132 caused a significant increase in ROS content with respect to the controls at both 30 min of incubation, when pollen tube emergence was initiated, (Figure 5A) and at 90 min, when a large number of tubes had already emerged and elongated in the control pollen (Figure 5B). This increase in intracellular ROS levels in response to MG132 treatment has been previously observed both in plants and animal cells [43]–[45]. ROS are well known to play a dual role as both deleterious and beneficial species depending on their concentration in plants. At high concentrations ROS causes damage to biomolecules, whereas at low/moderate concentrations it acts as a second messenger in intracellular signaling cascades that mediate several responses in plant cells. ROS levels play an important role in pollen tube emergence and elongation and must be strictly regulated [46], [47]. Thus, ROS over-accumulation induced by proteasome inhibition may contribute to the observed inhibition of pollen germination and tube elongation.

Interestingly, the inhibition of the proteasome resulted in the accumulation of oxidatively-modified polypeptides, as revealed by an increase in carbonylated proteins (Figure 5C). This could be the consequence of the increased intracellular ROS levels as well as the impaired degradation of oxidatively damaged proteins, due to proteasome inactivation. Moreover, MG132 led to the accumulation of an isoflavone reductase-like protein (ISFR), however qPCR data indicate that this accumulation is associated with an increased gene expression (Figure 2), suggesting that the proteasome could affect protein levels indirectly. Indeed, it has been demonstrated that ISFR transcript levels are upregulated by oxidants [48], [49], further confirming a role of proteasome inhibition in modulating ROS homeostasis.

In the current analysis, MG132 treatment led also to the accumulation of a protein disulfide isomerase-like (PDI). PDI facilitates the retrotranslocation of some misfolded proteins from the ER to the proteasome in yeast and animal cells [50], [51]. Immunogold labeling with anti-ubiquitin antibody has provided direct proof of UbP accumulation in ER membranes upon MG132 treatment, leading to significant ER stress [13]. In addition, PDI is recognized and selectively degraded by the proteasome [52]. Thus, the inhibition of the proteasome may contribute to alter PDIcellular homeostasis, which has been shown to be important for pollen tube growth in *Arabidopsis thaliana* [53].

In conclusion, the accumulation of ATP in pollen treated with MG132 indicates that the inhibition of tube growth is not due to a deficit in energy power. Our data indicate that the increase in the content of ATP is due to a lower usage rather than an increased production.

**Figure 6:**
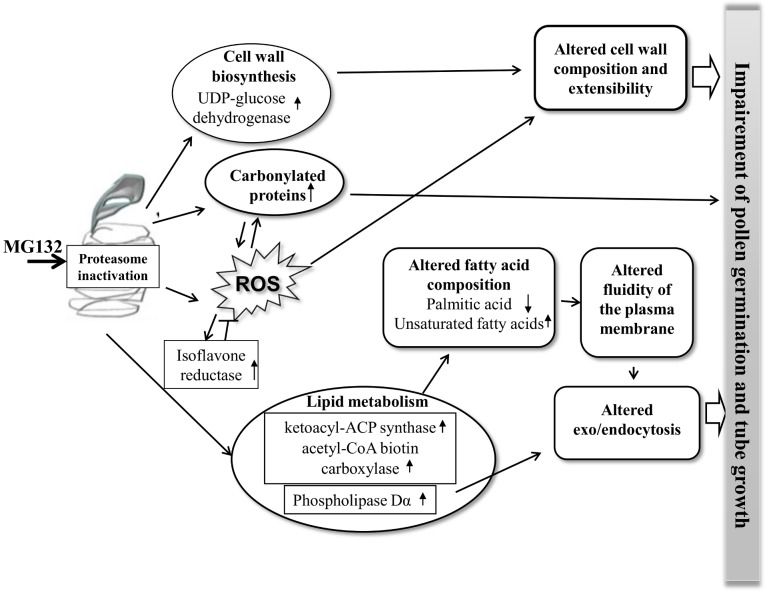
Model of the response of kiwifruit pollen to MG132 treatment. The proposed mechanisms suggest that ROS-induction, lipid metabolism and cell wall biosynthesis are important cellular events in pollen tube development under MG132 treatment. Only some of the responsive proteins are indicated. Arrows “↑” and “↓” indicate an increasing or a decreasing amount of proteins and metabolites.

As shown in Figure 6, proteasome inhibition affects the accumulation of some proteins involved in lipid metabolism (ketoacyl-ACP synthase, acetyl-CoA biotin carboxylase and phospholipase Dα) and induces the alteration of the composition of fatty acids. This could lead to a change in the fluidity of the plasma membrane and to the impairment of the exo/endocytosis during the elongation of the pollen tube. The ROS increase in MG132-treated pollen disrupts the redox homeostasis as indicated also by the induction of the isoflavone reductase. This fact could increase the level of oxidatively damaged proteins as well as the level of the impaired degradation due to proteasome inhibition. Moreover, the intracellular ROS accumulation and the altered UDP-glucose dehydrogenase homeostasis could modify the composition of the cellular wall and extensibility, contributing in this way to the altered growth and morphology of the pollen tube.

## Supporting Information

Table S1 **Blast analysis.**
(DOC)

Table S2 **Primers for qPCR analysis.**
(DOC)

Table S3 **Differentially expressed proteins in samples treated with MG132 with respect to the DMSO-control identified by LC-ESI-MS/MS analysis.**
(DOC)
