# Supplementary material for: Proteomic Analysis of MG132-Treated Germinating Pollen Reveals Expression Signatures Associated with Proteasome Inhibition
Source: PLoS One. 2014 Sep 29;9(9):e108811. doi: 10.1371/journal.pone.0108811 (PMC4181863; doi:10.1371/journal.pone.0108811)
Supplement: Table S1 — Blast analysis. (DOC) [file pone.0108811.s001.doc]

| **Spot** | **Protein identified by Mascot** | **Query ID**  **(EST of Actinidia)** | **RefSeq ID**  **(species, length in nucleotides) retried by BLAST using Actinidia** | **Score (Max-Total)** | **Query cover** | **E value** | **Identities** |
| --- | --- | --- | --- | --- | --- | --- | --- |
| **1, 2** | gi|225455555  Enolase  *Vitis Vinifera* | [FG518186.1](http://www.ncbi.nlm.nih.gov/nucleotide/195271344?report=genbank&log$=nucltop&blast_rank=2&RID=UGKMCF14014) | XM_002283596.1  *Vitis vinifera* | 822-822 | 100% | 0.0 | 88% (627/714) |
| **3,4,5** | gi|3914394  Phosphoglycerate mutase *Mesembryanthemum crystallinum* | FG459194.1 | XM_002266169.1 *Vitis vinifera* | 691-691 | 100% | 0.0 | 87% (533/612) |
| **6,7** | gi|2499497  Phosphoglycerate kinase  *Nicotiana tabacum* | FG490494.1 | XM_002263914.2  *Vitis vinifera* | 628-628 | 97% | 0.0 | 83% (577/693) |
| **8** | gi|4033417  Inorganic Pyrophosphatase  *Hordeum vulgare* | FG527876.1 | XM_004165231.1  *Cucumis sativus* | 606-606 | 68% | 2e-177 | 85% (499/585) |
| 9 | gi|315258127  UDP-glucose 6-dehydrogenase-like  *Galega orientalis* | FG502351.1 | XM_002269656.2  *Vitis vinifera* | 481-481 | 99% | 4e-133 | 84%  (413/489) |
| **10** | gi|210110274  Ketoacyl-ACP synthase  *Arachis hypogaea* | FG427830.1 | XM_002272838.2  *Vitis vinifera* | 470-470 | 74% | 1e-129 | 83%  (428/513) |
| **11,12** | gi|226425233  Biotin carboxylase 1  *Arachis hypogaea* | FG515662.1 | XM_002266453.1  *Vitis vinifera* | 446-446 | 96% | 4e-129 | 83%  (413/498) |
| **13** | gi|195984445  phospholipase D alpha (PLD)  *Gossypium raimondii* | FG501800.1 | XM_002268159.1  *Vitis vinifera* | 761-761 | 99% | 0.0 | 86%  (617/718) |
| **14** | gi|255537515  Aminopeptidase N-like  *Ricinus communis* | FG410667.1 | XM_003631365.1  *Vitis vinifera* | 411-715 | 95% | 4e-118 | 86% (332/387) |
| **15** | gi|568839552  T complex protein 1*  *Citrus sinensis* | FG528103.1 | XM_002524583.1  *Ricinus communis* | 797-797 | 84% | 0.0 | 86%  (633/734) |
| **16** | gi|566176847  Lysyl-tRNA synthetase  *Populus trichocarpa* | FG472187.1 | XM_002269548.2  *Vitis vinifera* | 623-623 | 99% | 6e-176 | 87%  (478/548) |
| **17,18,19** | gi|147779855  Initiation factor 4A-11*  *Vitis vinifera* | FG465889.1 | XM_002278083.2  *Vitis vinifera* | 765-765 | 98% | 0.0 | 88%  (564/639) |
| **20** | gi|255548505  Disulphide isomerise  *Ricinus communis* | FG426404.1 | XM_003521619.1  *Glycine max* | 483-483 | 95% | 2e-133 | 81%)  (504/623) |
| **21** | gi|149349524  isoflavone reductase-like protein 4 (IFRL4)  *Clarkia breweri* | FG409932.1 | NM_001281018.1  *Vitis vinifera* | 398-398 | 93% | 7e-108 | 79%  (483/615) |
