# Supplementary material for: Proteomic Analysis of MG132-Treated Germinating Pollen Reveals Expression Signatures Associated with Proteasome Inhibition
Source: PLoS One. 2014 Sep 29;9(9):e108811. doi: 10.1371/journal.pone.0108811 (PMC4181863; doi:10.1371/journal.pone.0108811)
Supplement: Table S2 — Primers for qPCR analysis. (DOC) [file pone.0108811.s002.doc]

| **Table S2. Primers for qPCR analysis.** | | | | | |
| --- | --- | --- | --- | --- | --- |
| **Protein** | **Spot** | **mRNA accession Actinidia** | **Homolog (%) *** | **Primers** | **Exons (size of intron)**  **Amplicon (bp)** |
| Enolase | 1, 2 | FG518186.1 | XM_002283596.1  (88%) | F:TCTTCCAGTGGGAGCTTCTAC  R:GCAAAGCCACCTTCATCACC | 8-9 (714)  132 |
| Phosphoglycerate mutase | 3, 4, 5 | FG459194.1 | XM_002266169.1  (87%) | F:TACGTGAGAAAGGTGTTGATGC  R:TGAGGGGCTTCACCAAGAAC | 4-5 (474)  133 |
| Phosphoglycerate kinase | 6, 7 | FG490494.1 | XM_002263914.2  (83%) | F:TCGGACCTGATTCCATCAAGG  R:GTTGTCACCCCCTTGCCAC | 6-7 (330)  160 |
| Inorganic Pyrophosphatase | 8 | FG527876.1 | XM_004165231.1  (88%) | F:CATGCAGGAACCAGTTCTTCC  R:TTCACCCTGATCAATCATAGGCA | 5/6-7 (80+70)  79 |
| UDP-glucose dehydrogenase | 9 | FG502351.1 | XM_002269656.2  (84%) | F:GTGAACCGTGTTGTTGCCTC  R:AAGGCAAACCCCAAAACAGC | (same exon)  71 |
| Ketoacyl-ACP synthase | 10 | FG427830.1 | XM_002272838.2  (83%) | F:GAAGGCGCTTGAAGATGCG  R:CACACCAGCTCGCTCCTTAT | 1-2 (1840)  70 |
| Biotin carboxylase | 11,12 | FG515662.1 | XM_002266453.1  (89%) | F:GGCGAAGCACCAAGTAGTCA  R:GCATTCTCGGCAAGGAAACC | 2-3 (1584)  113 |
| Phospholipase Dα | 13 | FG501800.1 + FG489344.1 | XM_002268159.1  (86 + 83%) | F:AAGCGGAGAATATGAACCATCAG  R:GACCTCTGGTTGATGTTGGC | 3-4 (262)  156 |
| Aminopeptidase N-like | 14 | FG410667.1 | XM_003631365.1  (86%) | F:GGGAGCCGAAGTTGTCAGG  R:GCATCTCGCATGGCAGCA | 20-21 (100)  135 |
| T complex protein 1 | 15 | FG528103.1 | XM_002524583.1  (86%) | F:TCTTCACTTGGCCCTGTTGG  R:CTCAGCCAACTCAACAAGCAC | 2-4 (192+106)  141 |
| Lysyl-tRNA synthetase | 16 | FG472187.1 | XM_002269548.2  (87%) | F:CCCTCCGCAAACAACAACTC  R:GCCAACGGACTCATTATCTCTGG | 13-14 (2155)  114 |
| Initiation factor 4A-11 | 17,18,19 | FG465889.1 | XM_002278083.2  (88%) | F:AGTCACTTCGCCCTGATTACAT  R:GCAGAGAACACCCCAACCTG | 3-4 (350≈)  136 |
| Disulphide isomerase | 20 | FG426404.1 | XM_003521619.1  (81%) | F:TGCCCTTGCTGAGTTTGTGAA  R:TGCAATGGCCGCACCATG | 4-5 (449)  161 |
| Isoflavone reductase-like protein 4 | 21 | FG409932.1 | NM_001281018.1  (79%) | F:GCGCCCCCCAGAGATAAAG  R:TGATCGTGTAGGTACCAATGTCAT | 3-4 (249)  91 |
| Actin 11 | - | FG436985.1+ FG473703.1 | AT3G12110.1 | F:GTGTGCGATAATGGAACTGGAATG  R:CCATGCCAACCATCACACCA | 6-7 (330)  118 |
| 18S rRNA | - | FG447958.1 | AT2G01010 | F:AATCCTGACACGGGGAGGTA  R:ACCAGACTTGCCCTCCAATG | (same exon)  118 |

Detailed informations about primer design are reported in material and method. * The accession of the closest homolog with an available genomic sequence and its homology to Actinidia’s sequence is indicated.
