# Supplementary material for: Proteomic Analysis of MG132-Treated Germinating Pollen Reveals Expression Signatures Associated with Proteasome Inhibition
Source: PLoS One. 2014 Sep 29;9(9):e108811. doi: 10.1371/journal.pone.0108811 (PMC4181863; doi:10.1371/journal.pone.0108811)
Supplement: Table S3 — Differentially expressed proteins in samples treated with MG132 with respect to the DMSO-control identified by LC-ESI-MS/MS analysis. (DOC) [file pone.0108811.s003.doc]

| **Table S3. Differentially expressed proteins in samples treated with MG132 with respect to the DMSO control identified by LC-ESI-MS/MS analysis.** | | | | | | | | | | |
| --- | --- | --- | --- | --- | --- | --- | --- | --- | --- | --- |
| **Spot** | **Acc. N. ^a^** | **Protein description** | **Species** | **Loc.^b^** | **Score^c^** | **Pep./ cov. (%)^d^** | **MW/ pI Theor.^e^** | **MW/pI Exp.^e^** | **Anova^f^** | **Fold of var.^g^** |
| **Energetic Metabolism** | | | | | | | | | | |
| 1 | gi|225455555 | Enolase | *Vitis Vinifera* | C | 665 | 11/36 | 48.3/6.2 | 70.0/5.6 | 0.009 | + 1.5 |
| 2 | gi|225455555 | Enolase | *Vitis Vinifera* | C | 623 | 12/32 | 48.3/6.2 | 52.0/5.7 | 0.013 | + 1.6 |
| 3 | gi|3914394 | Phosphoglycerate mutase | *Mesembryanthemum crystallinum* | C | 282 | 7/14 | 61.3/5.4 | 66.0/5.5 | 0.017 | +1.7 |
| 4 | gi|3914394 | Phosphoglycerate mutase | *Mesembryanthemum crystallinum* | C | 309 | 8/20 | 61.3/5.4 | 66.0/5.7 | 0.001 | + 1.6 |
| 5 | gi|3914394 | Phosphoglycerate mutase | *Oryza sativa* | C | 436 | 11/21 | 61.0/5.2 | 66.0/5.6 | 0.004 | + 1.8 |
| 6 | gi|2499497 | Phosphoglycerate kinase | *Nicotiana tabacum* | C | 336 | 5/18 | 50.0/8.5 | 40.0/5.8 | 0.004 | + 2.2 |
| 7 | gi|2499497 | Phosphoglycerate kinase | *Nicotiana tabacum* | C | 101 | 2/7 | 50.0/8.5 | 40.0/5.6 | 9,640e-004 | + 1.9 |
| 8 | gi|4033417 | Soluble inorganic pyrophosphatase | *Hordeum vulgare* | C | 198 | 4/20 | 24.1/5.9 | 25.0/5.6 | 0.025 | + 1.5 |
| **Cell Wall** | | | | | | | | | | |
| 9 | gi|315258127 | Putative UDP-glucose dehydrogenase | *Galega orientalis* | C | 451 | 7/15 | 53.5/5.7 | 60.0/5.7 | 0.002 | + 1.7 |
| **Lipid Metabolism** | | | | | | | | | | |
| 10 | gi|210110274 | Ketoacyl-ACP synthase | *Arachis hypogaea* | Ch | 120 | 2/7 | 50.5/8.5 | 50.0/5.9 | 0.041 | + 1.5 |
| 11 | gi|226425233 | Biotin carboxylase | *Arachis hypogaea* | Ch | 168 | 5/12 | 58.8/7.6 | 50.0/5.6 | 0.016 | +1.7 |
| 12 | gi|226425233 | Biotin carboxylase | *Arachis hypogaea* | Ch | 292 | 7/19 | 60.0/6.9 | 48.0/5.9 | 0.008 | + 1.7 |
| 13 | gi|195984445 | Phospholipase Dα | *Gossypium raimondii* | Ch | 289 | 7/9 | 92.0/5.4 | 95.0/5.6 | 0.039 | + 1.7 |
| **Protein Synthesis** | | | | | | | | | | |
| 14 | gi|255537515 | Aminopeptidase | *Ricinus communis* | Ch | 146 | 3/3 | 98.0/6.0 | 95.0/5.7 | 0.016 | + 1.7 |
| 15 | gi|568839552 | T-complex protein 1 subunit beta-like * | *Citrus sinensis* | C | 660 | 10/27 | 57.1/5.6 | 63.0/5.9 | 0.027 | + 1.7 |
| 16 | gi|566176847 | Lysyl-tRNA synthetase | *Populus trichocarpa* | C | 186 | 4/7 | 68.3/5.9 | 66.0/5.8 | 0.039 | + 1.7 |
| 17 | gi|147779855 | Initiation factor 4A-11* | *Vitis vinifera* | C | 383 | 7/20 | 47.0/5.4 | 48.0/5.3 | 0.005 | + 2.3 |
| 18 | gi|147779855 | Initiation factor 4A-11* | *Vitis vinifera* | C | 766 | 17/38 | 47.0/5.4 | 48.0/5.2 | 0.003 | + 2.2 |
| 19 | gi|147779855 | Initiation factor 4A-11* | *Vitis vinifera* | C | 936 | 19/53 | 47.0/5.4 | 51/5.2 | 0.021 | - 1.7 |
| **Stress Response** | | | | | | | | | | |
| 20 | gi|255548505 | Disulfide isomerase | *Ricinus communis* | C | 189 | 4/14 | 39.6/5.4 | 38.0/5.7 | 0.016 | + 1.5 |
| 21 | gi|149349524 | Isoflavone reductase-like  protein 4 | *Clarkia breweri* | C | 270 | 6/16 | 34.0/5.4 | 35.0/5.8 | 0.001 | + 1.6 |
| a) NCBI accession number; b) Cellular localization: C, cytoplasm; Ch, chloroplast; c) Mascot score (http://www.matrixscience.com); d) number of unique peptides identified /protein sequences coverage; e) Theorical and experimental molecular weight/ Isoelectric point; f) protein expression Anova test value (<http://www.totallab.com/products/samespots/news>); g) protein expression fold variation in MG132 *vs* control ; *Sequence annotation derived from BLAST alignment against nr-NCBI database. | | | | | | | | | | |
